# Search is a verb: systematic review searching as invisible labor

**DOI:** 10.5195/jmla.2021.1226

**Published:** 2021-07-01

**Authors:** Amanda Ross-White

**Affiliations:** 1 amanda.ross-white@queensu.ca, Associate Librarian, Bracken Health Sciences Library, Queen's University, Kingston, ON, Canada

**Keywords:** authorship, librarians, information storage and retrieval, systematic reviews as topic, feminism, work

## Abstract

Invisible labor is a term used by labor economists to describe work that contributes, and is often even necessary, to the economy but largely goes unrecognized and unpaid. Despite the fact that systematic review searching is a significant task for many librarians and knowledge professionals, the search process can be considered a form of invisible labor because it often goes without recognition. This occurs sometimes through not granting authorship to the librarian who performed the intellectual contribution of search development and sometimes through a devaluing of the search process by the choice of language used to describe the search. By using the term search as a passive verb or noun, authors devalue the real intellectual labor involved in searching, which includes decisions related to search terms and combinations, database selection, and other search parameters. This commentary explores the context of how searching is described through the concept of invisible labor.

## INTRODUCTION

Systematic review searching, or knowledge synthesis searching, is one task that librarians and other knowledge professionals receive remuneration for as part of their paid employment. However, because much of this labor is hidden, either from the primary authors of systematic reviews, funders, or institutional administrators, search has become a form of invisible labor. By remaining unseen, systematic searchers risk having their contributions to the review process being undervalued. One of the critical ways academics demonstrate value and make their work visible is through authorship. Yet even the language used to describe the search process betrays the value (or lack thereof) placed on that process. When searching is described as an active verb, with human agency as part of the process, the work of the searcher is given more value. When used as a passive verb or as a noun, the role of human agency is reduced, making the work performed by the searcher invisible. When the act of searching is reduced to “applying an algorithm,” connotations to computer science and programming further reduce the role of human agency in the process and devalue the work and potentially the remuneration of those engaged in this labor. This commentary will examine the context of how searching is described in systematic reviews through the concept of invisible labor.

## WHAT IS INVISIBLE LABOR

Invisible labor is often used to describe the work done primarily by women in the home to keep it functioning and running well, but that largely goes unrecognized and unpaid, despite contributing to the broader economy [1]. Earlier conceptions of invisible labor characterized it as something which is not considered legitimate work *because* it goes unpaid. As Daniels describes it in her groundbreaking essay, “any effort even if it is arduous, skilled, and recognized as useful—perhaps essential—is still not recognized as work if it is not paid”[2]. This creates an unvirtuous circle. If any task that goes without pay is not work, then we can continue to exploit the worker by choosing not to see what they are doing as work and in turn not pay for it. For Daniels, who viewed this concept with a feminist lens, the

understanding of the essential characteristic of work is that it is something for which we get paid. This idea is associated with activity in the public world, which is dominated by men and separated from those private worlds of family and personal ships where women predominate. There may be exchanges in households and friendships but they are not monetary. Even activity in the public sphere, such as volunteering and community service, it is not work if it isn't paid. However, any activity we do for pay wherever it is found, even if we enjoy it, must, by definition be work [2].

This feminist view of invisible labor was more common when the division between paid labor done outside the home and unpaid labor done inside the home was more strictly delineated by gender.

However, invisible labor can also refer to

activities that occur within the context of paid employment that workers perform in response to requirements (either explicit or implicit) from employers and that are crucial for workers to generate income, to obtain or retain their jobs, and to further their careers, yet are often overlooked, ignored and/or devalued by employers, consumers, workers and ultimately the legal system itself [1].

Invisible work can refer both to the tasks being done and the persons performing these tasks. Consider the invisible labor behind a banana. We know that in order for bananas to cost a few cents per pound, they had to arrive at the store in a truck, where an invisible worker unloaded the truck in the early hours of the morning. The truck driver picked up the bananas at a warehouse, likely located in an industrial area, which is isolated from the rest of the city. Because I live in North America, there is no way the bananas were produced locally, so they arrived at the warehouse in a large container ship, from a country like Ecuador, where we give little thought to the workers on the large banana plantations. All these types of work are a form of invisible labor because they are done by a workforce that remains largely unseen and with little thought from the end consumer of the banana.

With COVID-19 pandemic restrictions encouraging many of us to work from home, we are also beginning to see for the first time many of the additional types of labor imposed on workers that we have always taken for granted, such as requiring workers to present themselves in a particular way, dress in a certain style, or wear makeup (often required of women, although not always explicitly). This too is invisible labor and is often invisible even to the worker themselves. Workers in many industries, but especially those that are reliant on tips, are often expected to perform this type of invisible labor by matching the standards of appearance set by the brands they work for and investing their own time and money in wearing the right clothes, hairstyles, and makeup in a way that maximizes income. This type of invisible labor is also often viewed through a feminist framework as expectations of appearance and dress code are often applied more strictly for women than men [3, 4].

Other invisible labor that has come into view as more employees work from home includes the emotional labor involved in building relationships with coworkers, clients, and others. Time spent in conversation before meetings and engaging with others in breakrooms or through travel from one location to another is not wasted time but rather the invisible labor of the social context of work.

## INVISIBLE LABOR AND LIBRARY WORK

That library work, and in particular, search, is an invisible form of labor is an idea that needs to be considered, particularly in the context of remote work. Daniels described the mental load of the housewife as one form of invisible work. In her words:

planning, restocking, improvising, and adapting to family quirks and demands require effort that the housewives themselves do not recognize as work; they say they cannot understand why they become so tired or use so much time in making the effort [2].

For systematic reviews and other forms of knowledge synthesis, the work of the reference interview or question development is a similar physical, cognitive, and emotional effort. Particularly when dealing with a student or novice reviewer who may have little experience in framing a research question, considerable time and effort can be expended by the librarian who needs to find ways to manage expectations, frame the question in an answerable way, and elucidate the real question being asked—a form of invisible labor that is often poorly reflected in reference statistics as traditionally captured by most libraries. Many reference statistics count only “basic” and “complex” questions, or some variation on this schema, reducing what might be a very time-consuming and labor-intensive interaction to a single number [5]. If this reference conversation is imbued with power dynamics, as can often be the case with students, faculty, and librarians, there may be other forms of invisible labor involved as well. For example, where there may be disagreement between the faculty researcher and the librarian about the nature of the review, with a graduate student seeking guidance from both persons, a librarian may need to both educate the student and do so in a way that does not disrespect the faculty member or create further confusion for the novice reviewer. Navigating these social complexities is also a form of invisible labor.

Certainly, the concept that a reference interview is a form of invisible work is not new. Ehrlich and Cash described how reference work is invisible labor because a librarian is not merely there to “'connect people and raw data but to help them approach, consider, and make sense of information” [4]. The invisible background work of understanding how and where to search is critical to the labor being performed. When this observation was made over twenty years ago, when unmediated online search was still relatively new, Ehrlich and Cash posited that this vital role for librarians was likely to become even more hidden. Building trust in your skills as an intermediary was important, with trust being “established through reputation, branding, personal acquaintance, and observations/testing of the intermediaries' behavior over a period of time”[6].

With search, an activity many librarians perform daily in some capacity, whether as systematic review searchers or not, the challenge then becomes changing our mental model about what constitutes work so that our own beliefs about the value of search do not become undermined due to familiarity. While systematic review authors may not value search because they do not perform search, librarians are at risk of devaluing search because they perform it daily.

## SEARCH DESCRIPTIONS IN SYSTEMATIC REVIEWS

The issue of librarians as authors in systematic reviews has been previously considered [7–9]. While authorship in academic papers is the ultimate achievement in visibility, at least within the health sciences, there are strict criteria for who can be considered an author. According to the International Committee of Medical Journal Editors (ICMJE) requirements, authors must make:

- substantial contributions to the conception or design of the work; or the acquisition, analysis, or interpretation of data for the work, AND
- drafting the work or revising it critically for important intellectual content, AND
- final approval of the version to be published, AND
- agreement to be accountable for all aspects of the work in ensuring that questions related to the accuracy or integrity of any part of the work are appropriately investigated and resolved [10].

If a contributor meets any one of these criteria, without meeting all four, they are to be named in the acknowledgments. Yet, we can determine from our own experience and from reading published systematic reviews that this is not often the case. Among systematic reviews published by researchers at Queen's University, only 37% named a librarian as an author or in the acknowledgments [7]. More troubling, at least from the perspective of search as a form of invisible labor, is that 9% explicitly stated that a librarian was involved in performing the search yet did not name the individual at all.

This proportion is consistent with Wislar et al., who found approximately 12% of articles in high-impact medical journals had a ghost author (that is, someone who did work meeting the ICMJE criteria for authorship but was not named as an author) [11]. However, Gulen et al. looked specifically at Cochrane systematic reviews and found that only 2% of reviews had ghost authors [12]. This discrepancy is likely due in part to the different definitions of ghost authorship used in the two studies, as both used similar surveys of the corresponding author. For example, Gulen et al. used only those who fulfilled all ICMJE criteria, which would exclude any contributors who were not given the opportunity to provide approval of the final. They acknowledged this discrepancy by looking at acknowledgments, where they found that “13% of the reviews did not acknowledge a person despite their contribution to the work” [12].

This is also consistent with Lariviere et al.'s bibliographic study of authors in nine disciplines, which found that the persons who actually performed the experiments were least likely to be granted authorship [13]. In this analysis, persons who performed work that was perceived as technical (as opposed to conceptual) were less likely to be considered authors and often had less experience than the more senior authors who performed the conceptual tasks. In a similar manner, some systematic review authors perceive searching as a technical task that involves insufficient intellectual contribution to merit authorship.

In analyzing the description of search in systematic reviews, we can see there are several ways to describe a search: as an active or passive verb or as a noun. The use of search as an active verb (i.e., “Five major databases … were searched by a health librarian to find articles discussing the relationship between social factors and recovery after a hip fracture” [15]), provides agency on the part of the searcher, as opposed to the use of search as a passive verb, where no human is mentioned (i.e., “papers published between 1980 and 2014 in the English language were searched” [14]). Search as an active verb turns it into an intellectual task with judgment calls and decisions being made on keywords and databases chosen and not chosen, degree of sensitivity and specificity, and other determinations in which the searcher relies on education and experience to make decisions. The use of the passive voice, or search used as a noun, minimizes the role of the person performing the task. Some examples of the passive voice when describing the search include: “A systematic review of the literature was performed in November 2017, with a repeat and expanded search conducted in July 2019” [16], or “Search strategies combining indexing keywords relevant to CBT-I in primary care (Box 1) were used with each database. The initial search included articles published from January 1987 until 16 November 2017” [17]. Examples of active searches include: “We conducted keyword-based searches in PubMed, PsycINFO, Cochrane, CINAHL, and Embase databases” [18], or “We searched both MEDLINE and EMBASE for relevant studies using the following key words” [19]. Many articles use both passive and active descriptions of the search, such as one that references the work done by librarians but does not name them: “The search was current as of September 2016. The specific search strategy was developed by a Health Sciences librarian with expertise in systematic review searching with input from the project team. The search strategy was then reviewed by a second Health Sciences librarian” [19].

We need also consider the role of gender when analyzing who is named as an author or contributor and whose work is made visible. When Macaluso et al. analyzed gender by contributions, women were more likely to have performed the technical work of performing the experiments and were also less likely to receive authorship [20]. Given that librarianship is both a female-dominated profession (and medical librarianship even more so, at 88% female), this has implications for how our work is viewed in the context of invisible labor [21]. If search is perceived as a female job, with all the invisible labor implications that go with that, does this put search in the same category as many other instances of invisible labor, particularly within the domestic sphere? What does it mean when we replace the vocabulary of librarianship (search) with the more male-dominated language of computer science (algorithm)?

In this context of searching as a form of invisible labor, advocating for coauthorship where warranted and named acknowledgment in other instances is important work beyond the personal impact on one's career. By ensuring that the invisible labor of performing search is recognized by systematic review authors, librarians are able to demonstrate value to the reviewers who often lack awareness of the intellectual work involved in developing a question for review and translating it into a systematic search. Further, authorship and acknowledgment provide a tangible measure to other areas of academia, in particular to library and university administrators, who lack familiarity with the work involved and therefore often do not provide recognition for this work. Making the invisible visible is a crucial task for ensuring that this too is seen as work. If work is a “purposeful human activity involving physical or mental exertion that is not undertaken solely for pleasure and has economic or symbolic value,” then how we perceive and conceptualize that work impacts us in real economic terms [1]. Naming this as work, for which we are entitled to appropriate credit and remuneration, has value.
